# Revelation of ZnS Nanoparticles Induces Follicular Atresia and Apoptosis in the Ovarian Preovulatory Follicles in the Catfish* Mystus tengara* (Hamilton, 1822)

**DOI:** 10.1155/2016/3927340

**Published:** 2016-03-08

**Authors:** Nilanjana Chatterjee, Baibaswata Bhattacharjee

**Affiliations:** ^1^Department of Zoology, Ramananda College, Bishnupur, Bankura 722122, India; ^2^Department of Physics, Ramananda College, Bishnupur, Bankura 722122, India

## Abstract

Important physicochemical characteristics of water like dissolved oxygen content, pH, and so forth were found to change in a dose dependent manner, showing a negative correlation with the nanoparticle concentration, when ZnS nanoparticle (NP) was exposed to water. This observation could be attributed to the enhanced photooxidation property associated with ZnS in its NP form. Under this situation, the catfish* Mystus tengara* was forced to live in hypoxia in its habitat. This condition was found to hamper the natural oogenesis process of the fish. Due to exposure at relatively lower concentration of ZnS NPs (250 *μ*g/L), most of the maturing follicles of* M. tengara* failed to complete the process of vitellogenesis properly and underwent preovulatory atresia followed by oocytic apoptosis. For relatively higher concentration of ZnS nanoparticles (500 *μ*g/L), the previtellogenic process continued with increasing number of apoptotic cells; however the vitellogenic process was found to be totally blocked. This unusual reproductive behaviour in female* M. tengara* can be attributed to the decreased metabolism of the fishes under ZnS nanoparticle induced hypoxia.

## 1. Introduction

Exposure of nanoparticles (1–100 nm) in different water bodies is gradually becoming a menacing concern for aquatic milieu. Nanoparticles that get into the water bodies from various domestic, natural, and industrial sources often end up with serious consequences for the aquatic organisms [1–5]. They affect the aquatic life either directly by entering into the biological system or indirectly by altering the water physicochemical parameters and creating noxious stress in the organisms [6–12].

The ZnS nanoparticles (NPs) have shown to exhibit some changes in the physicochemical parameters of the water bodies such as dissolved oxygen level and pH, which in turn regulates the water quality and hence affects the aquatic fauna [10–12]. Being an integral part of the water bodies, fishes will most likely be affected by the consequences of the dissolved ZnS NPs. It has now been emphasized in the last two decades that atresia can be related to the programmed cell death: apoptosis, in order to maintain the tissue level homoeostasis in the multicellular organisms [13]. Various factors such as cytokines, hormones, viruses, xenobiotics, radiation, oxidative stress, and hypoxia can induce apoptosis, after atresia [14]. As ZnS NPs reduce the level of dissolved oxygen content in water, fish are forced to face hypoxia [9–12] and consequently the oxidative stress in their habitat. So, it is likely that the maturing oocytes will come across prescheduled follicular atresia followed by apoptosis under exposure to ZnS NPs.

The experiment was conducted with an aim to show that the ZnS NPs when released into the water bodies from different sources may end up in creating some physiological alterations and changes in the reproductive behaviour in the aquatic animals such as* Mystus tengara*. This is prevalently inedible fish in eastern India and Bangladesh and hence any adverse change in its reproductive behaviour will create a negative impact on the commercial fish market. Therefore, systematic study is needed to monitor the hazardous effect of ZnS NPs on the female reproductive system of* M. tengara*. Further, this work is also aimed to establish the fact that such NPs demand proper treatment before they are released into the water bodies.

## 2. Methodology

### 2.1. Preparation and Characterization of ZnS Nanoparticles

The ZnS NPs are prepared by simple wet chemical method using zinc nitrate hexahydrate [Zn(NO_3_)·6H_2_O] as zinc precursor and sodium sulphide (Na_2_S) as sulphur precursor [5, 9]. The as precipitated nanoparticles were filtered out and were washed for several times in distilled water and absolute alcohol (100% ethanol) and then were dried at 30°C in a vacuum oven. The dried nanoparticles were then treated with ultrasonic irradiation for 10 minutes to obtain uniform mono dispersed suspension required for several characterization processes. The nanoparticles were characterized using X-ray diffraction study (XRD), Transmission Electron Microscopy (TEM), Particle Size Analysis (PSA), Energy dispersive X-ray study (EDX), and X-ray Photoelectron Spectroscopy (XPS). The synthesis process and characterization results have been discussed in detail elsewhere [5, 9].

### 2.2. Fish Husbandry

Matured live female* M. tengara* (*n* = 60) of average length of 14 ± 3.5 cm and weight of 33 ± 6.0 g were collected from local fishermen of Bankura district, West Bengal, India, in their late growth phase (February) for two consecutive years (2011-12). After collection, fishes were kept in water tight containers (capacity of 100 litres) containing tap water that has been allowed to stand for a few days. Fishes are maintained at 25°–35°C of temperature to ensure the natural environment. Small, regular supplies of food were provided to the fishes.

After 10 days of collection, fishes were divided into three groups each having population of 20 fishes. After that, two groups were exposed to two different concentrations of ZnS nanoparticles (250 *μ*g/L and 500 *μ*g/L) in addition to the controlled one at the onset of maturation phase, that is, early March. The alterations in the hepatic and ovarian histology for two different concentrations of ZnS NPs were studied with respect to that of the controlled population.

### 2.3. Histological Study

The controlled and treated fish were sacrificed; the ovaries and liver were dissected out and subjected to routine histological procedures. Upgrading or dehydration of tissues was done by putting them for 10 minutes each (2 changes) in distilled water, 30%, 50%, 70%, and 90% ethanol, and finally absolute alcohol (100% ethanol). Dehydration using upgraded alcohol was followed by xylene treatment, paraffin embedding (melting point 56°–58° Celsius), section cutting (4 *μ*m), and staining using Delafield's Haematoxylin and Eosin (HE), Iron-Alum Haematoxylin (IA), and Mallory's triple (MT) stain for the ovaries and HE for the liver tissues.

### 2.4. Histometric Study

The diameter of the hepatocytes is measured at four different points in the cell with the help of an ocular and reticle micrometer (having the lowest precision scale of 2 *μ*m) attached to the light compound Olympus microscope for about 50 different randomly selected cells and then their standard deviation (SD) is calculated. The number of different oocytic follicles is analysed and counted based on the area wise cell counting method on the tissue surface observed under the aforesaid microscope.

### 2.5. Selection of ZnS NP Concentration

The fishes were exposed to ZnS NPs by dissolution of the NPs into water in specified concentrations. ZnS NPs of a fixed size (average diameter of 3 ± 0.5 nm) were selected for our experiment and the threshold concentration for observing the effect was recognised as 250 *μ*g/L. This effect was found to saturate for* M. tengara* beyond the concentration of 500 *μ*g/L (Figure 10). Hence, the toxicity test was designed for the above-mentioned two concentrations of ZnS NPs for 60 days.

## 3. Results and Discussion

### 3.1. Impact of ZnS Nanoparticles on Physicochemical Properties of Water

Due to the enhanced surface photooxidation property associated to the greater surface area, ZnS in the nanoparticle form pose serious threat to the aquatic lives by reducing the dissolved oxygen content in the water [5]. The oxygen dissolved in water bodies is crucial for the organisms and creatures living in it. As the amount of dissolved oxygen drops below normal levels in water bodies, the water quality is impaired and creatures living in it suffer from hypoxia and oxidative stress.

The photooxidation of the surface of ZnS NPs using the dissolved oxygen of water under sunlight and consequent reduction of dissolved oxygen content in water has been confirmed from detailed study of S 2p core level X-ray photoelectron spectra of ZnS nanoparticles after different time of exposures [9]. During the surface photooxidation process of ZnS NPs, the S atoms exposed to the ZnS surface got oxidized and an increase in concentration of chemisorbed SO_2_ at ZnS surface with increasing exposure time was observed in the samples [9]. The oxide leaves the surface as a molecular species (SO_2_), leaving Zn and a freshly exposed layer of ZnS behind. Water may dissolve a part of the SO_2_ released in the process causing reduction in the pH value of the water [10]. Subsequently under the exposure of ZnS NPs, the aquatic fauna of that particular habitat was forced to live in an oxygen depleted and acidified atmosphere [9–12].

### 3.2. Histological and Histometric Studies

**Figure 1:**
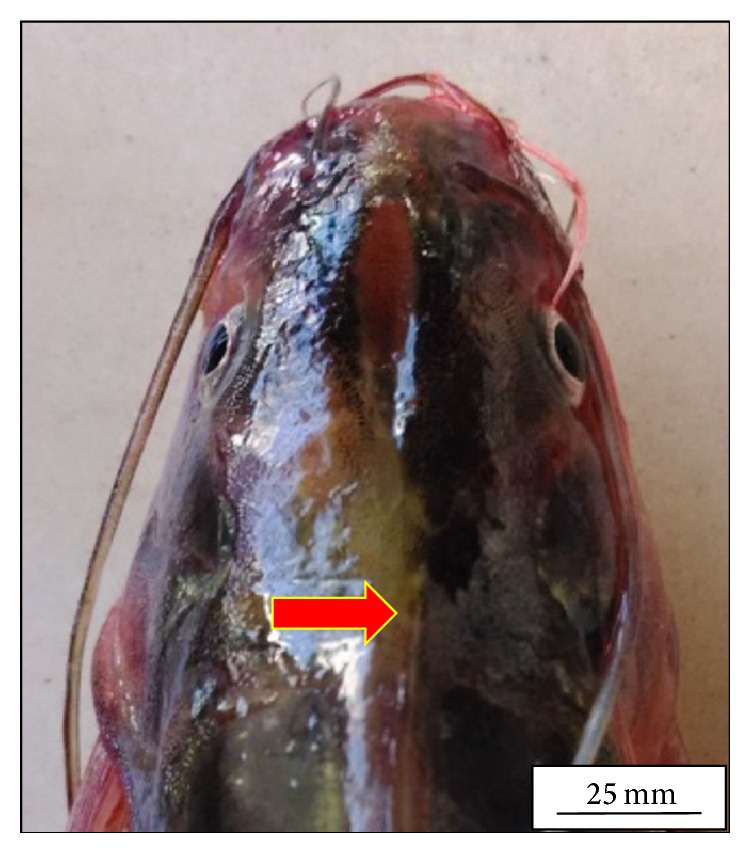
Dorsal view of the head of* Mystus tengara* showing the posterior extremity of the posterior fontanel (solid red arrow) which extends up to one-third of the supra occipital bone in the adult fish.

**Figure 2:**
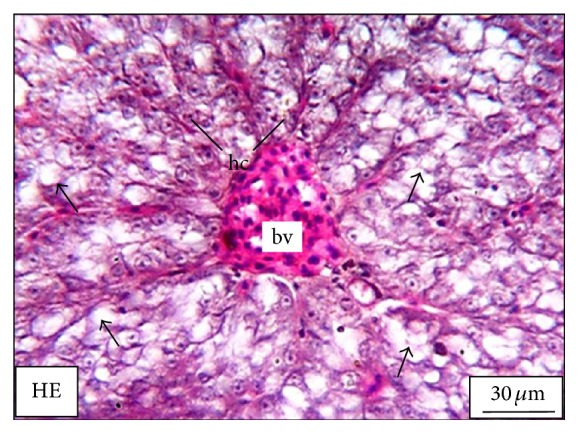
The histological section of the liver of a mature adult* Mystus tengara* during the spawning phase (June) showing large reserve of fat cells (small black arrow) and enlarged hepatocytes bearing prominent nuclei (hc). The section also shows a ramified blood vessel (bv) at the centre of the tissue under controlled condition [HE: Haematoxylin-Eosin stain].

The adult* M. tengara* can be identified morphologically by the characterized posterior extremity of the fontanel which extends up to one-third of the supraoccipital bone in the head (Figure 1). Morphologically it can be distinguished from its closest congener,* M. vittatus* and other striped* Mystus* of the Ganga-Brahmaputra basin [15].  Figure 2 shows the hepatic histology of* M. tengara* kept under controlled condition. In this figure, liver cells are found to be large with regular outlines. These cells are dominated by storage deposits chiefly composed of lipid and glycogen. Depending upon the amount of intracellular lipid and glycogen, individual hepatocytes display significant variations in their appearance within HE stained sections. The nuclei are found to be large and eccentrically located indicating the normal condition of the cells having cellular size of 14 ± 0.4 *μ*m. At a relatively low ZnS NPs concentration (250 *μ*g/L) (Figure 5), the hepatocytes are found to slightly diminish in size (12 ± 0.4 *μ*m) with respect to that of the fish under controlled condition (14 ± 0.2 *μ*m). The sinusoidal spaces are found to increase for the treated fish due to lack of granulated cytoplasmic contents, denoting a slight fall in the metabolic rate. The fat vacuoles lessen in number and gradually decrease in size due to utilization of some of the energy reserves by the fish under study. This denotes a state of partial physiological dormancy, since the rate of metabolism is reduced and at the same time the fish utilizes its stored energy. Under exposure to relatively higher ZnS NPs concentration (500 *μ*g/L) (Figure 9), the hepatocytes grow even smaller in size (10 ± 0.2 *μ*m), with more conspicuous empty spaces and spatially disrupted hepatic chords. These observations can be attributed to the relatively slower metabolic rate and consequent break up or cataclysm of the hepatocytes chiefly due to apoptosis or programmed cell death (cellular debacle) under the adverse metabolic conditions caused by environmental hypoxia.

**Figure 3:**
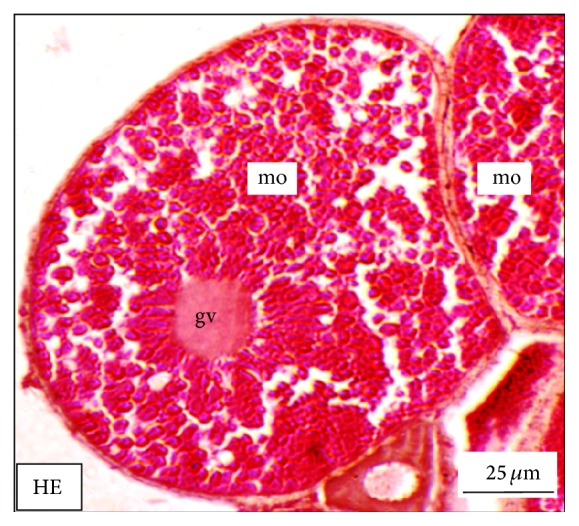
The histological section of the ovary of a mature adult* Mystus tengara* during the spawning phase (June) revealing the yolk laden mature ovum (mo) with laterally migrating germinal vesicle [HE: Haematoxylin-Eosin stain].

**Figure 4:**
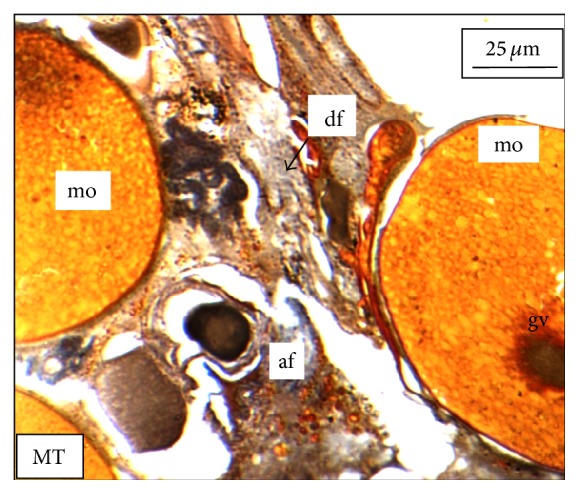
The histological section of a mature ovary of* M. tengara* during the spawning phase (June) showing the presence of mature ovum (mo), atretic follicle (af), and discharged follicle (df) under controlled condition [Mallory's triple stain].

**Figure 5:**
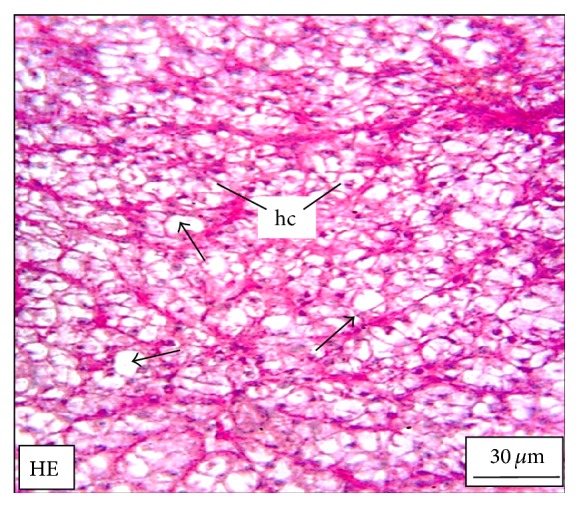
The histological section of the liver of a* M. tengara* during the early spawning phase (June) depicting comparatively fewer numbers of fat vacuoles (small black arrows) and slight decrease in the size of the hepatocytes (hc) under experimental condition when the tissues are treated with ZnS nanoparticles at the concentration of 250 *μ*g/L [HE: Haematoxylin-Eosin stain].

**Figure 6:**
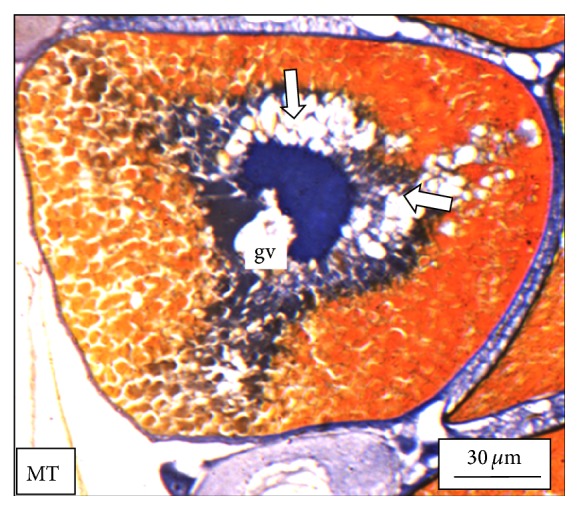
The histological section of the ovary of a* M. tengara* during the early spawning phase (June) showing maturing follicles (mf) with impeded yolk deposition. Empty yolk vesicles (solid white arrows) around the basophilic germinal vesicle (gv) under experimental condition when the tissues are treated with ZnS nanoparticles at the concentration of 250 *μ*g/L also support the deposition of the yolk material centripetally [Mallory's triple stain].

**Figure 7:**
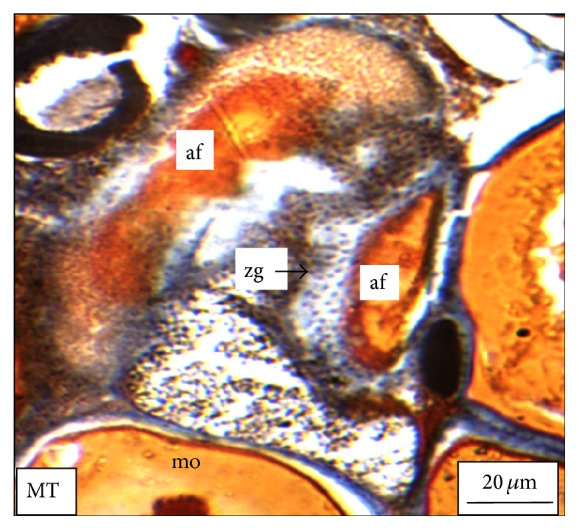
The histological section of the ovary of a* M. tengara* during the early spawning phase (June) showing mature oocytes undergoing apoptosis and showing liquefaction of the yolk materials along with hypertrophy of the granulosa cell (zg) [HE: Haematoxylin-Eosin stain].

**Figure 8:**
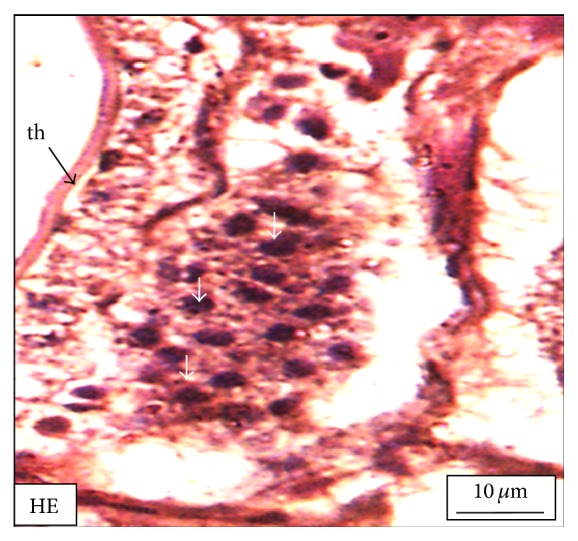
The histological section of the ovary of a* M. tengara* during the early spawning phase (June) showing an apoptotic mature follicle (mf) bearing conspicuous pycnotic nuclei (white arrows) of the zona granulosa layer and intact theca layer (th) when the tissues are treated with ZnS nanoparticles at the concentration of 250 *μ*g/L [Mallory's triple stain].

**Figure 9:**
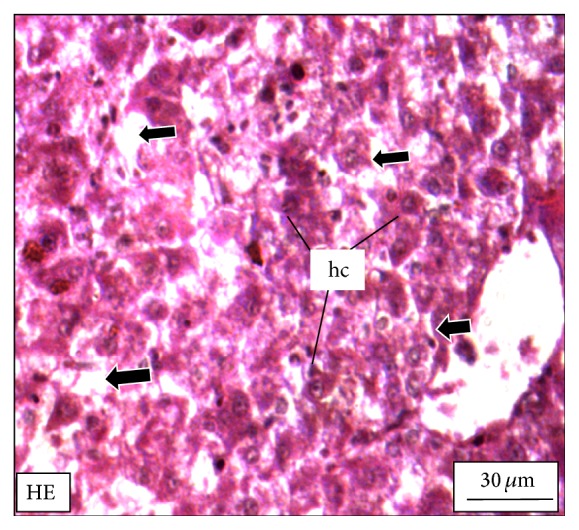
The histological section of the liver of a* M. tengara* during the late spawning phase (August) depicting the diffused array of hepatocytes (hc) that is diminished in size but bearing prominent nuclei. The fat vacuoles are totally absent; instead some conspicuous empty spaces (black solid arrows) are generated in between the hepatocytes when the tissues are treated with ZnS nanoparticles at the concentration of 500 *μ*g/L [HE: Haematoxylin-Eosin stain].

**Figure 10:**
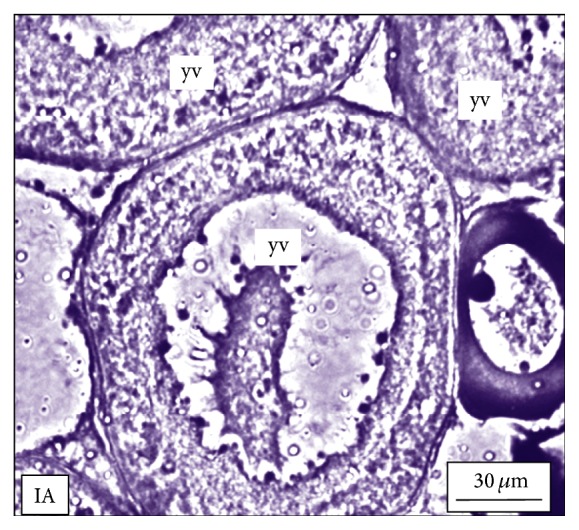
The histological section of the ovary of a* M. tengara* during the late spawning phase (August) displaying the yolk vesicle (yv) stages of oocyte without any trace of yolk deposition when the tissues are treated with ZnS nanoparticles at the concentration of 500 *μ*g/L [IA: Iron-Alum Haematoxylin].

**Figure 11:**
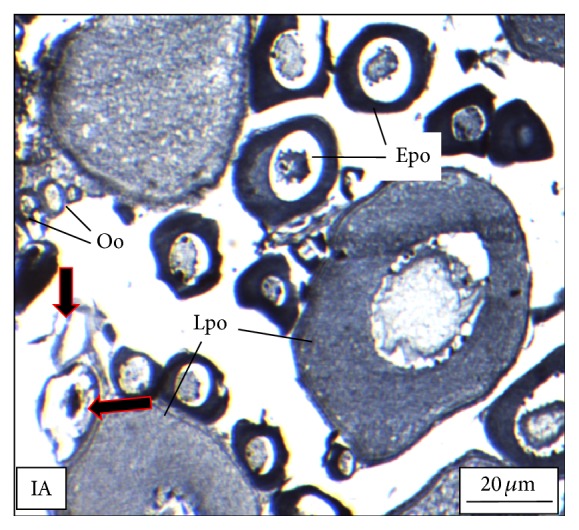
The histological section of the ovary of a* M. tengara* during the late spawning phase (August) showing all of the previtellogenic oocytic stages [Oo: oogonia, Epo: early perinucleolar oocyte, and Lpo: late perinucleolar oocyte] with few apoptotic follicles (af) that are gradually merging with the ovarian stroma when the tissues are treated with ZnS nanoparticles at the concentration of 500 *μ*g/L [IA: Iron-Alum Haematoxylin].

**Figure 12:**
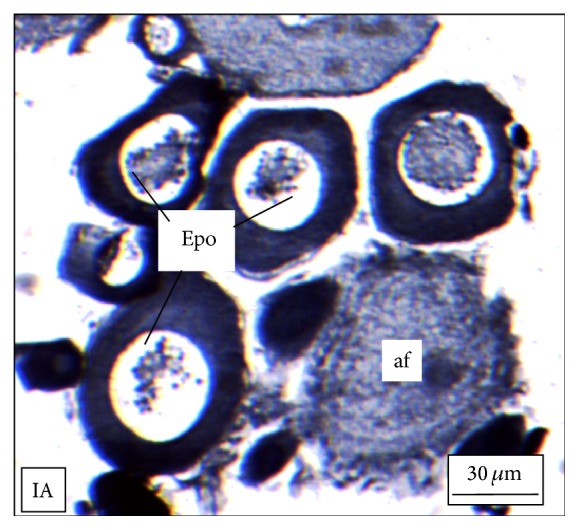
The histological section of the ovary of a* M. tengara* during the late spawning phase (August) showing large number of early perinucleolar oocytes (Epo) and few previtelline atretic follicles (af) when the tissues are treated with ZnS nanoparticles at the concentration of 500 *μ*g/L [IA: Iron-Alum Haematoxylin].

Under controlled condition, more than 80% of the ovaries were found to be occupied with yolk laden matured ovum with eccentrically placed germinal vesicle (Figure 3). Many discharged follicles and few atretic follicles were also noticed with matured ova (Figure 4). After exposure to a relatively low concentration (250 *μ*g/L), about 60% of the ova were found with maturing follicles with delayed yolk deposition (Figure 6). A large number of the maturing oocytes are observed to undergo atresia, which are again of two different types, hypertrophic (Figure 7) and cystic (Figure 8). Ovarian follicular atresia firmly mentions the failure of a follicle to rupture or ovulate. Further, follicular atresia comprises the fate or demise of all follicles except those destined for ovulation. Though follicular atresia is a common phenomenon in teleosts [16, 17], cystic atresia in fish oocytes is comparatively a rare event [18]. In the present study, atresia is followed by follicular apoptosis prior to the ovulation process, hence reducing the probable number of discharged follicles. At a relatively higher ZnS NP concentration (500 *μ*g/L), the atretic follicles are found to develop solely from the previtelline stages (Figures 11 and 12) and there is hardly any visible yolk deposition stage, mature oocyte, and discharged follicle during the spawning period when the fishes are sacrificed.

The observed alterations in histology were probably because the fishes abnormally reduced the metabolic activity due to their exposure to lower dissolved oxygen content in the water as a result of the photooxidation of the ZnS NPs. Photooxidation of the ZnS NPs, which consumed the dissolved oxygen content of water [9], leads to the formation of SO_2_ and ZnO. ZnO forms a film on the water surface that acts as a barrier for the surface or atmospheric oxygen which would otherwise have supplied the fish with oxygen for the respiratory purpose. Due to dissolution of some part of SO_2_ in it, the pH of the water has also been recorded to decrease [11, 12]. The reduction in water pH was found to increase with the increasing concentration of dissolved ZnS NPs which leads to the metabolic acidosis of the fishes. Under such adverse situation, the fish reduces the rate of ATP consumption by avoiding the process of vitellogenesis and hence prefers to undergo follicular atresia. The vitellogenic follicles when splits during atresia probably act as a rich source for energy to the fish undergoing hypoxia.

In case of the hypertrophic atresia the cells of the zona granulosa become highly atrophied and engulf almost the entire yolk meal. Besides, the biological apoptotic signals imbalance of certain physical forces may also be held responsible for such follicular atresia in the vitellogenic oocytes. The intracellular pressure which is the net outcome of the summation of the osmotic pressure, fluid pressure, and the pressure exerted by the accumulating yolk materials balances the pressure from the extracellular environment. However, due to less supply of the yolk materials from the hepatocytes [19], a fall in pressure is experienced in the cell interior with respect to the cell exterior; this probably acts as a cue for the zona granulosa cells to increase their size and invade inside the oocytes for apoptosis. However, for the cystic atresia that has been detected from the previtellogenic as well as the vitellogenic oocytes, therefore it is clear from our study that the ZnS NPs play a contributing role in follicular atresia and apoptosis.

## 4. Conclusion

The ZnS in its bulk form is generally regarded as nontoxic when exposed to water. However, when ZnS NPs are exposed to water, their enhanced photooxidation property makes them harmful to the aquatic environment by creating a condition of hypoxia in the environment. Due to exposure at relatively lower concentration of ZnS NP (250 *μ*g/L), most of the maturing follicles of* Mystus tengara* failed to complete the process of vitellogenesis properly and underwent preovulatory atresia followed by oocytic apoptosis. Under exposure to a relatively higher concentration of ZnS NPs (500 *μ*g/L), the previtellogenic process continued with increasing number of apoptotic cells; however the vitellogenic process was found to be totally blocked in the oocyte maturation process of* M. tengara.* This unusual reproductive behaviour in female* M. tengara* can be attributed to the decreased metabolism of the fishes under ZnS NP induced hypoxia.

Two remedial measures are suggested from our end to make the water free from the detrimental effect of ZnS NPs. Firstly, removal of ZnS NPs from the industrial and household effluents by blocking them in the terrestrial zone by means of “paedological sieve” and, secondly, conversion of the ZnS NPs into their bulk counterpart by employing processes such as thermal agglomeration, before they are released into the water bodies. In this manner we can pave the way for a “cleaner earth and safer future” for the aquatic organisms.
